# Antagonizing miR-218-5p attenuates Wnt signaling and reduces metastatic bone disease of triple negative breast cancer cells

**DOI:** 10.18632/oncotarget.12593

**Published:** 2016-10-12

**Authors:** Hanna Taipaleenmäki, Nicholas H. Farina, Andre J. van Wijnen, Janet L. Stein, Eric Hesse, Gary S. Stein, Jane B. Lian

**Affiliations:** ^1^ Department of Cell Biology, University of Massachusetts Medical School, Worcester, MA, USA; ^2^ Heisenberg-Group for Molecular Skeletal Biology, Department of Trauma, Hand & Reconstructive Surgery, University Medical Center Hamburg-Eppendorf, Hamburg, Germany; ^3^ Department of Biochemistry & Cancer Center, University of Vermont College of Medicine, Burlington, VT, USA; ^4^ Department of Orthopedic Surgery, Mayo Clinic, Rochester, MN, USA; ^5^ Department of Anatomy and Cell Biology, Indiana University School of Medicine, Indianapolis, IN, USA

**Keywords:** metastasis, breast cancer, osteolysis, Wnt signaling, miR-218-5p

## Abstract

Wnt signaling is implicated in bone formation and activated in breast cancer cells promoting primary and metastatic tumor growth. A compelling question is whether osteogenic miRNAs that increase Wnt activity for bone formation are aberrantly expressed in breast tumor cells to support metastatic bone disease. Here we report that miR-218-5p is highly expressed in bone metastases from breast cancer patients, but is not detected in normal mammary epithelial cells. Furthermore, inhibition of miR-218-5p impaired the growth of bone metastatic MDA-MB-231 cells in the bone microenvironment *in vivo.* These findings indicate a positive role for miR-218-5p in bone metastasis. Bioinformatic and biochemical analyses revealed a positive correlation between aberrant miR-218-5p expression and activation of Wnt signaling in breast cancer cells. Mechanistically, miR-218-5p targets the Wnt inhibitors Sclerostin (SOST) and sFRP-2, which highly enhances Wnt signaling. In contrast, delivery of antimiR-218-5p decreased Wnt activity and the expression of metastasis-related genes, including bone sialoprotein (BSP/IBSP), osteopontin (OPN/SPP1) and CXCR-4, implicating a Wnt/miR-218-5p regulatory network in bone metastatic breast cancer. Furthermore, miR-218-5p also mediates the Wnt-dependent up-regulation of PTHrP, a key cytokine promoting cancer-induced osteolysis. Antagonizing miR-218-5p reduced the expression of PTHrP and Rankl, inhibited osteoclast differentiation *in vitro* and *in vivo*, and prevented the development of osteolytic lesions in a preclinical metastasis model. We conclude that pathological elevation of miR-218-5p in breast cancer cells activates Wnt signaling to enhance metastatic properties of breast cancer cells and cancer-induced osteolytic disease, suggesting that miR-218-5p could be an attractive therapeutic target for preventing disease progression.

## INTRODUCTION

Breast cancer is among the most prevalent malignancies worldwide, constituting a tremendous medical and socio-economic problem [1]. After initial treatment distant metastases frequently occur after years or even decades of a disease-free survival [2]. Bone is a primary site for breast cancer metastases and about 70% of breast cancer patients at an advanced stage of the disease suffer from osteolytic bone metastases, a stage at which the disease is incurable [2]. Osteolytic metastases are often associated with debilitating bone pain and skeletal-related events (SREs), including pathological fractures. Currently, patients are treated with anti-resorptive drugs (bisphosphonates or denosumab, a monoclonal antibody against the Receptor activator of nuclear factor kappa-B ligand (RANKL)) that restrict the progression of bone destruction and increase survival [3]. However, novel therapeutic targets are needed for intervention prior to bone metastasis.

Bone metastases perturb the physiological maintenance of bone mass and bone remodeling by the coordinated activities of matrix-producing osteoblasts and bone-resorbing osteoclasts. Cancer cells produce growth factors such as Parathyroid hormone-related protein (PTHrP), which stimulate osteoblasts to secrete RANKL and other bone–resorbing cytokines [4]. RANKL increases osteoclast activity and subsequent bone degradation during which matrix-derived growth factors, e.g. Transforming Growth Factor-β1 (TGF-β1), are released into the metastatic micro-environment. These factors further stimulate cancer cell proliferation creating a ‘vicious cycle’, a multi-directional process that perpetuates metastatic bone destruction [5].

Small non-coding microRNAs (miRNAs) have established functions in both carcinogenesis and bone remodeling [6–8]. A large pool of evidence suggests that miRNAs are potential targets for therapeutic intervention of cancer [9, 11, 12]. By silencing the expression of hundreds of genes simultaneously, a single miRNA can act as an epigenetic master regulator of important biological processes. Deregulation of the miRNA-dependent control also contributes to oncogenesis and leads to the loss of tumor suppressors and upregulation of oncogenes, thus ultimately driving tumor progression and metastasis [9, 12]. Global profiling and genome wide sequencing have identified transcription factors and miRNAs that are deregulated in tumor cells [13]. For example, compromised control of epithelial-mesenchymal transition (EMT) and mesenchymal-epithelial transition (MET) by perturbation of miRNA-transcription factor networks is well documented in cancer progression [14]. Furthermore, abnormal expression of the bone-specific transcription factor Runx2 in bone metastatic cells [15] is in part due to the loss of two miRNAs targeting Runx2 and the BMP pathway [16]. Thus, while supporting normal development of tissues, miRNAs also mediate the progression of metastatic bone disease [11].

Wnt signaling represents another important pathway that regulates normal tissue formation, as well as contributes to tumor onset and progression [17, 18]. Osteogenesis is highly dependent on the presence of Wnt activators and the absence of Wnt inhibitors [19]. miR-218 was previously characterized to promote bone formation by osteoblasts through upregulation of Wnt signaling [20]. Here, we investigated functional consequences of high levels of miR-218-5p in breast cancer cells. We hypothesized that aberrant miR-218-5p expression would induce the osteomimetic properties of breast cancer cells and cause aggressive metastatic bone disease. Our cellular and *in vivo* studies revealed a positive correlation of miR-218-5p expression and β-catenin signaling in bone metastases and demonstrate that miR-218-5p targets two inhibitors of Wnt signaling, sclerostin (SOST) and secreted frizzled related protein (SFRP2). Furthermore, we show a striking inhibition of tumor growth in the bone and a reduced osteolytic disease with antimiR-218-5p blockade of Wnt–dependent activation of genes related to both metastasis and osteolysis and elucidated the underlying mechanism of antimiR-218-5p in bone metastatic breast tumors. In response to antimiR-218-5p, tumor growth in the bone marrow microenvironment and the accompanying metastatic bone disease was largely inhibited. These findings may have translational potential for therapeutic intervention to reduce bone metastasis by inhibiting miR-218-5p in breast cancer cells.

## RESULTS

### miR-218-5p is increased in bone metastases and promotes breast cancer cell proliferation

**Figure 1:**
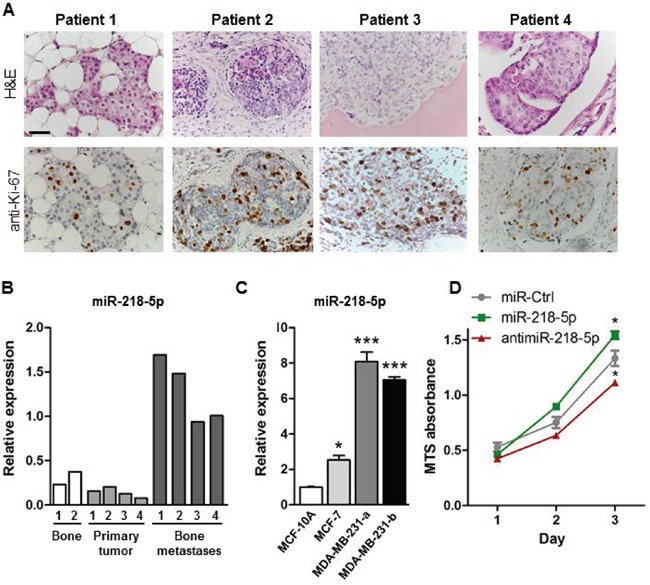
miR-218-5p is elevated in bone metastases **A.** H&E staining (upper panel) and immunohistochemical analysis of the proliferation marker Ki-67 (lower panel) in bone metastases from breast cancer patients. Scale bar indicates 50 μm. **B.** Expression of miR-218-5p was determined in healthy human bone (white bars), primary breast tumors (light grey bars) and bone metastasis biopsies obtained from breast cancer patients (dark grey bars) by qRT-PCR. **C.** miR-218-5p expression was analyzed in non-malignant epithelial MCF-10A cells, non-metastatic MCF-7 breast cancer cells and in two sub clones of metastatic MDA-MB-231 breast cancer cells by qRT-PCR. N= 4 independent experiments. Mean values ± SEM, * p<0.05, *** p<0.001 vs. MCF-10A. **D.** Cell proliferation was determined in MDA-MB-231 cells after transfection with miR-218-5p, antimiR-218-5p, or non-targeting control (miR-Ctrl) using an MTS Assay. N= 4independent experiments. Mean values ± SEM, * p<0.05 vs. miR-Ctrl.

To investigate the relevance of miR-218-5p in the context of bone metastases in humans, we examined the expression of miR-218-5p in healthy bone, primary breast cancer and bone metastases obtained from breast cancer patients. H&E staining and immunohistochemical analysis confirmed that all samples of metastatic tissue consisted of actively proliferating breast cancer cells (Figure 1A). As expected from previous studies [20], miR-218-5p was detected in healthy control bone (Figure 1B). Similarly, miR-218-5p was expressed in primary breast tumors, however, expression strikingly increased in bone metastases (Figure 1B). This finding was consistent with *in vitro* expression analysis of miR-218-5p in several breast cancer cell lines (Figure 1C). Expression of miR-218-5p was low in non-malignant, ER- epithelial MCF-10A cells and in early-stage, non-metastatic ER+ MCF-7 breast cancer cells and significantly increased in two sublines of ER- metastatic MDA-MB-231 breast cancer cells that grow aggressively in bone (Figure 1C). To confirm that miR-218 is linked to bone metastatic capacity rather than hormone-receptor status, expression was examined in the ER-negative MCF10 series of cell lines [21, 28, 29]. miR-218-5p expression was increased in pre-malignant MCF-10AT1 cells compared to non-malignant epithelial MCF-10A cells (Supplementary Figure S1). Importantly, expression was further increased in MCF10CA1, which have the ability to metastasize and grow in bone [22]. The specific high abundance of miR-218-5p in bone metastases in patients and bone metastatic breast cancer cells suggests that miR-218-5p contributes to the aggressive properties of metastatic breast cancer cells.

To functionally test this hypothesis, we modulated miR-218-5p levels in MDA-MB-231 cells by stable overexpression or inhibition of miR-218-5p using lentiviral vectors containing Green Fluorescent Protein (GFP; Supplementary Figure S2A). In addition, because viral-free delivery of small RNAs is more relevant for future translational applications, we tested the mechanistic function of miR-218-5p in breast cancer cells using a synthetic miR-218-5p mimic, inhibitor, and non-targeting control oligonucleotides (Supplementary Figure S2B). Delivery of miR-218-5p mimic significantly increased breast cancer cell proliferation while antagonizing miR-218-5p resulted in a reduced growth of metastatic cancer cells *in vitro* (Figure 1D). These results were corroborated by forced expression of miR-218-5p or its corresponding antimiR-218-5p in MDA-MB-231 cells (Supplementary Figure S2C). However, neither delivery nor forced expression of miR-218-5p mimic or inhibitor affected cell migration or invasion (Supplementary Figure S3A, S3B). These data suggest that miR-218-5p specifically supports cell proliferation and metastatic tumor growth in metastatic breast cancer cells.

### AntimiR-218-5p treatment reduces tumor growth in the bone marrow environment and protects bone from breast cancer-induced osteolytic disease

**Figure 2:**
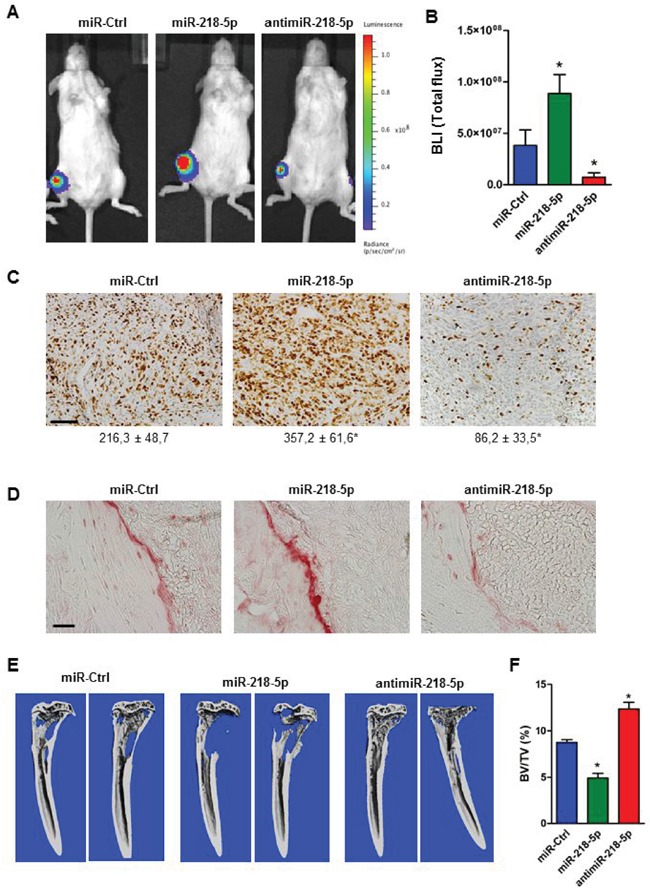
AntimiR-218-5p treatment reduces tumor growth and protects from breast cancer-induced osteolytic disease **A.** MDA-MB-231 cells stably expressing luciferase were transfected with the indicated oligonucleotide and transplanted into the tibiae of immunocompromised mice. Tumor growth was visualized after four weeks by bioluminescence imaging. **B.** Quantification of bioluminescence intensity (BLI) using Living Image software. N= 6 mice/ group. Mean values ± SEM, * p<0.05 vs. miR-Ctrl. **C.** Actively proliferating cancer cells were stained with Ki-67 and quantified with the Osteomeasure System. Scale bar indicates 50 μm. Number of Ki-67-positive cells/ field is indicated below each representative image. N= 6 mice/ group. Mean values ± SEM, * p<0.05 vs. miR-Ctrl. **D.** Presence of active osteoclasts in tumor bearing bones following miR-218-5p deregulation was visualized by TRAP staining. Scale bar indicates 50 μm. **E.** Osteolytic lesions were determined by μCT imaging and **F.** analysis of bone mass. BV/TV; bone volume/tissue volume. N= 6 mice/ group. Mean values ± SEM, * p<0.05 vs. miR-Ctrl.

To translate the *in vitro* findings into a preclinical model of osteolytic disease, we transfected MDA-MB-231 cells stably expressing luciferase (MDA-MB-231-*Luc*) with miR-218-5p mimic, antimiR-218-5p inhibitor, or the non-targeting control and injected tumor cells into the tibiae of immunocompromised SCID mice. Weekly bioluminescence imaging documented a significant increase in tumor growth when miR-218-5p mimic was delivered in MDA-MB-231*-luc* cells. In striking contrast, antimiR-218-5p reduced tumor growth in the bone marrow microenvironment (Figure 2A, 2B, Supplementary Figure S4). Supporting these observations, the number of actively proliferating tumor cells greatly increased by miR-218-5p while antagonizing miR-218-5p significantly impaired breast cancer cell proliferation *in vivo* (Figure 2C). Intratibial tumor growth was accompanied with increased osteoclast activity and bone resorption in the presence of miR-218-5p as demonstrated by intense TRAP staining in the bone-tumor interface (Figure 2D). Inhibiting miR-218-5p was highly effective in reducing osteoclast resorptive activity (Figure 2D) and provided protection from the development of osteolytic lesions as determined by μCT analysis (Figure 2E, 2F). Although the majority of the mice in all groups developed lung metastases (miR-Ctrl: 5/6, miR-218-5p: 6/6, antimiR-218-5p: 4/6 mice), antagonizing miR-218-5p reduced number and size of metastatic nodules in the lung thereby attenuating the overall metastatic burden (Supplementary Figure S5A-S5D). These data indicate that inhibition of miR-218-5p blocks the vicious cycle of osteolytic bone disease and the accompanying metastasis.

### Wnt signaling is enriched in miR-218-5p targets and activated in bone metastases

**Figure 3:**
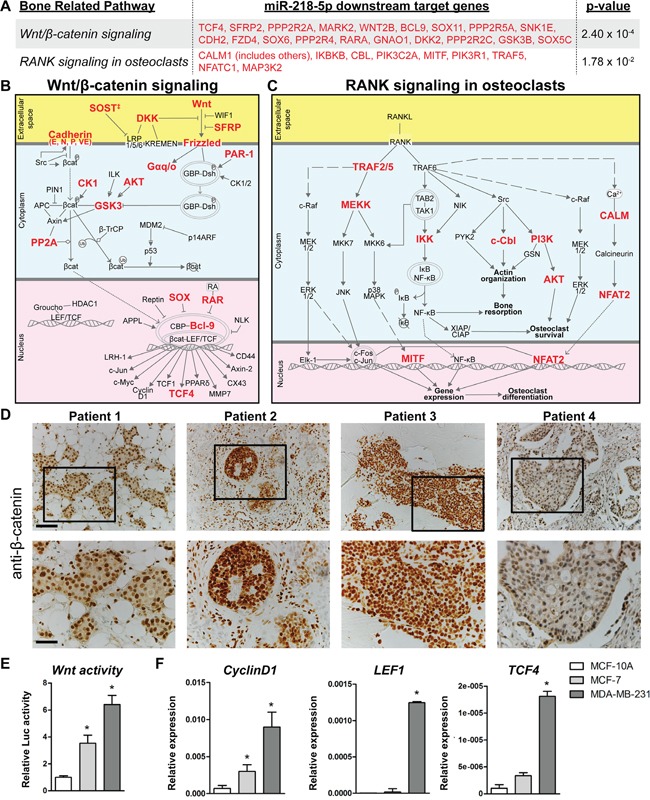
Wnt and RANK signaling were among the highest enriched pathways of miR-218-5p target genes **A-C.** Predicted and validated miR-218-5p targets, highlighted in red, are depicted in bone-related pathways. 116 Ingenuity canonical pathways were enriched over normal genomic distribution for the 967 downstream targets of miR-218-5p (p < 0.05). **A.** Target genes within the *Wnt/β-Catenin signaling* and *RANK signaling in osteoclasts* pathways are listed. The interaction partners and molecular mechanisms of these miR-218-5p target genes are depicted within the Wnt/β-Catenin signaling **B.** and RANK signaling **C.** pathways. ^‡^ SOST is not included in the Ingenuity Canonical Pathway Wnt/β-Catenin signaling and is not used to calculate the p-value in Figure 3A. SOST is well established to inhibit LRP5 [46]. **D.** Immunohistochemical analysis of β-catenin in bone metastasis biopsies. Scale bar indicates 100 μm (upper panel) and 50 μm (lower panel). **E.** Wnt signaling activity was determined by TopFlash assay in MCF-10A, MCF-7 and MDA-MB-231 cells. N= 3 independent experiments. **F.** Expression of Wnt target genes CyclinD1, LEF1 and TCF4 was analyzed by qRT-PCR and normalized to GAPDH expression. N= 3 independent experiments. Mean values ± SEM, * p<0.05 vs. MCF-10A.

To understand the underlying molecular mechanism of miR-218-5p in bone-metastatic breast cancer, we investigated the biological functions and pathways regulated by miR-218-5p. Using Ingenuity Pathway Analysis (IPA – www.ingenuity.com), 967 genes were identified as predicted or experimentally validated downstream targets of miR-218-5p. These 967 miR-218-5p target genes were significantly enriched for (p < 0.05) above normal genomic distribution in 116 Ingenuity canonical pathways. The essential bone-related pathways of *Wnt/β-catenin signaling* and *RANK signaling in osteoclasts* were identified in our analysis with 19 and 9 genes, respectively, being miR-218-5p targets (Figure 3A-3C).

Because Wnt signaling was highly enriched for in miR-218-5p targeted pathways and is tightly linked to breast cancer growth [30], we investigated the connection between Wnt signaling and miR-218-5p in bone metastases. Immunohistochemical staining of patient-derived bone metastasis biopsies revealed strong β-catenin staining in the nuclei of breast cancer cells (Figure 3D), supporting active engagement of Wnt signaling in proliferating bone metastases, which also highly express miR-218-5p (Figure 1A, 1B). In addition, Wnt signaling activity was significantly increased in metastatic MDA-MB-231 cells, which highly express miR-218-5p (Figure 1C) as demonstrated by the activity of the Wnt responsive TopFlash reporter and expression of Wnt target genes CyclinD1, LEF1 and TCF4 (Figure 3E, 3F). These results strongly suggest that miR-218-5p and Wnt signaling are connected in bone metastases.

### miR-218-5p and antimiR-218-5p regulate Wnt signaling through direct targeting of Wnt inhibitors with effects on cell proliferation

**Figure 4:**
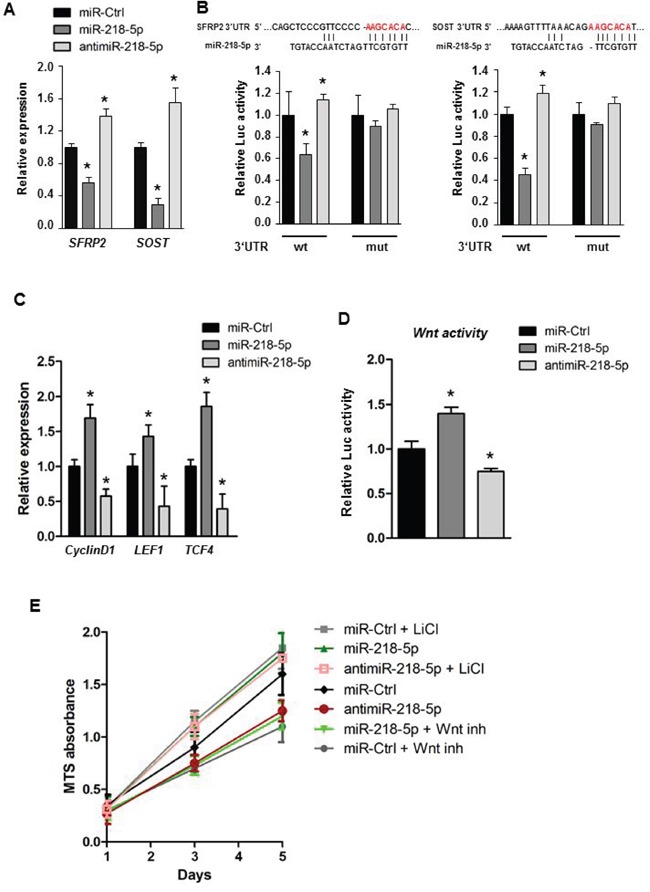
miR-218-5p directly targets Wnt inhibitors to augment Wnt signaling **A.** Gene expression analysis of Wnt inhibitors upon transfection with miR-218-5p or antimiR-218-5p oligonucleotides. Mean values ± SEM, * p<0.05 vs. miR-Ctrl. **B.** 3′UTR Luciferase assay was used to determine the direct binding between miR-218-5p and the 3′UTR of *SFRP2* or *SOST* (wt). The miR-218-5p target site (red letters) was mutated to alleviate binding. Mean values ± SEM,* p<0.05 vs. miR-Ctrl. **C.** Expression of the Wnt target genes CyclinD1, LEF1 and TCF4 was analyzedby qRT-PCR upon transfection with indicated oligonucleotides. Mean values ± SEM, * p<0.05 vs. miR-Ctrl.**D.** TopFlash luciferase assay to determine Wnt signaling activity in MDA-MB-231 cells after transfection with miR-218-5p or antimiR-218-5p oligonucleotides. Mean values ± SEM, * p<0.05 vs. miR-Ctrl. **E.** MTS cell proliferation assay of breast cancer cells transfected with miR-218-5p or antimiR-218-5p oligonucleotides. Wnt signaling was inhibited by a small molecule inhibitor CCT036477 (Wnt inh) or activated using Lithium Chloride (LiCl). GAPDH expression was used for qRT-PCR normalization. Mean values ± SEM, * p<0.05 vs. miR-Ctrl. N= 3-4 independent experiments.

We further examined the Wnt-miR-218-5p axis and direct miR-218-5p targets in breast cancer cells by focusing on Wnt inhibitors previously shown to be controlled by miR-218-5p in osteoblasts [20]. Consistent with results observed in osteoblasts, we found that the miR-218-5p reduced the expression of the endogenous sFRP2 whereas the antimiR-218-5p increased the expression level (Figure 4A). Furthermore, in metastatic cells, miR-218-5p downregulated Sclerostin expression, that was reversed by delivery of antimiR-218-5p (Figure 4A). Because the default activity of miRNAs is to repress protein translation by binding to the 3′UTR of mRNAs, we assessed whether miR-218-5p directly binds to SFRP2 and SOST using the 3′UTR reporter assays. Ectopic miR-218-5p significantly down-regulated Luciferase activity of both SFRP2 and SOST 3′UTRs in MDA-MB-231 cells (Figure 4B), while antimiR-218-5p increased luciferase activity via the 3′UTR of SFRP2 and SOST (Figure 4B). The regulation was blunted when the miR-218-5p binding sites in each of the potential target UTRs were mutated (Figure 4B). These findings indicate that the 3′UTRs of *SOST* and *SFRP2* are directly targeted by miR-218-5p to suppress their expression in metastatic breast cancer cells.

Stimulation of Wnt-signaling is predicted to activate the canonical Wnt responsive gene CyclinD1, which promotes breast cancer proliferation as well as LEF1 and TCF4, which are transcriptional mediators of the Wnt pathway. Indeed, CyclinD1, LEF1 and TCF4 were significantly increased in response to miR-218-5p delivery and decreased upon inhibition of miR-218-5p (Figure 4C). Furthermore, delivery of synthetic antimiR-218-5p oligos or lentivirus-mediated forced expression of antimiR-218-5p reduced Wnt signaling activity compared to control as shown by TopFlash assays that quantify Wnt-responsive promoter activity (Figure 4D, Supplementary Figure S6A). We also examined functional inhibition of Wnt signaling using a small molecule inhibitor, CCT036477, which reduced miR-218-5p –induced proliferation in MDA-MB-231 cells (Figure 4E). Furthermore, Wnt signaling activation by LiCl restored cell growth in antimiR-218-5p treated breast cancer cells (Figure 4E). Collectively, these data establish a functional relationship between Wnt signaling, miR-218-5p and breast cancer cell proliferation to promote tumor growth.

### Inhibition of miR-218-5p in breast cancer cells reduces Rankl expression in osteoblasts and impairs osteoclast differentiation

To better understand the anti-osteolytic function of miR-218-5p inhibition downstream of Wnt signaling, we performed gene expression analysis of Wnt-target genes related to bone metastasis and osteolysis. AntimiR-218-5p diminished the expression of several genes associated with bone metastasis and osteoclast activation, including osteopontin (OPN/SPP1), bone sialoprotein (BSP/IBSP), CXCR4, and PTHrP/PTHLH (Supplementary Figure S6B), suggesting that antimiR-218-5p suppresses the osteomimetic properties of metastatic breast cancer cells.

**Figure 5:**
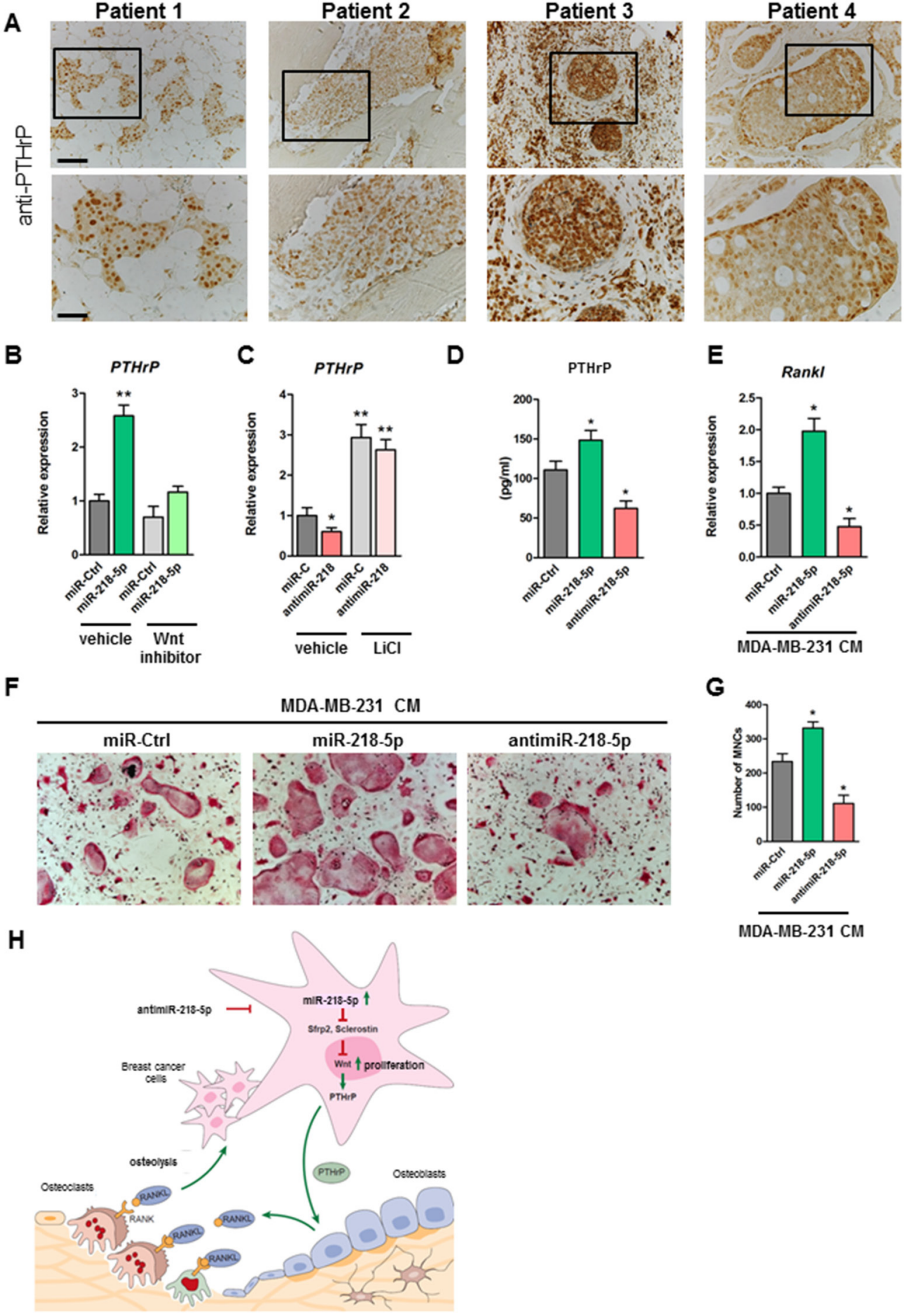
Inhibition of miR-218-5p in breast cancer cells decreases PTHrP expression, reduces Rankl in osteoblasts and impairs osteoclast differentiation **A.** PTHrP expression in bone metastases was determined by immunohistochemistry. Scale bar indicates 100 μm (upper panel) and 50 μm (lower panel). **B.** PTHrP mRNA expression was analyzed in MDA-MB-231 cells delivered with miR-218-5p upon inhibition of Wnt signaling using a small molecule inhibitor CCT036477. **C.** Cells were transfected with antimiR-218-5p and Wnt signaling was activated by Lithium Chloride. PTHrP mRNA expression was determined by qRT-PCR. **D.** Conditioned medium (CM) was collected from MDA-MB-231 cells transfected with miR-218-5p mimic or inhibitor and PTHrP protein level was determined using ELISA. **E.** Long bone osteoblasts were incubated with CM collected from miR-218-5p mimic or inhibitor -delivered MDA-MB-231 cells. Rankl expression was determined in osteoblasts by qRT-PCR. **F.** Osteoblast-osteoclast co-cultures were performed in the presence of CM from MDA-MB-231 cells transfected with miR-218-5p or antimiR-218-5p. **G.** Osteoclasts were stained with TRAP and TRAP-positive multinucleated cells (MNCs) were counted. **H.** Model of miR-218 function in the metastatic bone environment. Minimum of 3 independent experiments. Mean values ± SEM, *p<0.05, ** p < 0.01 vs. miR-Ctrl.

Since PTHrP is a well-established key cytokine promoting breast cancer-induced bone resorption and a recently identified Wnt target gene [4, 25], we focused on PTHrP as a novel cancer-related downstream effector of miR-218-5p. Immunohistochemical staining confirmed that PTHrP is highly expressed in bone metastases (Figure 5A). Functionally, miR-218-5p increased the expression of PTHrP in MDA-MB-231 cells, which was reduced upon Wnt inhibition (Figure 5B). Activation of Wnt signaling restored PTHrP expression in antimiR-218-5p treated cells, indicating Wnt-dependent regulation (Figure 5C). Given that PTHrP activates osteoclasts by inducing *Rankl* expression in osteoblasts, we collected conditioned medium (CM) from MDA-MB-231 cells transfected with miR-218-5p or antimiR-218-5p and measured the level of secreted PTHrP. Consistent with an activated gene expression, PTHrP protein concentration was increased in the CM upon miR-218-5p delivery and reduced when miR-218-5p was antagonized (Figure 5D). In support of our hypothesis, CM from cells delivered with miR-218-5p increased Rankl expression in long bone osteoblasts whereas Rankl expression was strikingly decreased when osteoblasts were cultured with CM from antimiR-218-5p-transfected breast cancer cells (Figure 5E). Consequently, CM from antimiR-218-5p treated cells reduced osteoclast differentiation in an osteoblast-osteoclast co-culture system as shown by the decreased TRAP staining and the reduced number of multinucleated cells (Figure 5F, 5G). These data suggest that antagonizing miR-218-5p in metastatic breast cancer cells impairs the osteolytic signaling cascades between breast cancer cells, osteoblasts and osteoclasts, which results in reduced osteolytic burden *in vivo*.

Together, our findings indicate that miR-218-5p is elevated in bone metastases and directly targets Wnt signaling inhibitors to increase Wnt signaling activity and promote breast cancer cell proliferation (Figure 5H). Furthermore, miR-218-5p increases the expression of PTHrP, resulting in an increased expression of Rankl in osteoblasts, an enhanced osteoclast differentiation and activated bone resorption. Importantly, antimiR-218-5p reverses these effects by reducing Wnt activity and expression of both PTHrP and Rankl, thereby attenuating the oncogenic and osteolytic properties of metastatic breast cancer cells.

## DISCUSSION

Our novel finding that miR-218-5p expression is significantly elevated in breast cancer cells that metastasize to bone provides strong support for a component of biological control that goes beyond regulation by transcription factors. Our results reinforce the concept that post-transcriptional miRNA regulators are aberrantly expressed in tumor progression and metastasis [10]. Moreover, our results indicate that the miR-218-5p-Wnt regulatory loop significantly contributes to the osteomimetic and metastatic properties of bone metastatic breast cancer cells. Abundance of miR-218-5p in MDA-MB-231 cells parallels function that mediated biological control in osteoblasts to increase Wnt signaling and expression of osteoblast related genes [20]. This finding suggests that miR-218-5p, which is highly expressed in bone metastases biopsies and metastatic cancer cells, may be a component of bone tumor growth and the accompanying osteolytic disease in patients. Related to this role of miR-218-5p, we found that antimiR-218-5p reduces expression of genes associated with osteolytic properties of cancer cells in bone, osteoclast activation and development of osteolytic lesions in mice. This finding positions antimiR-218-5p treatment as a potential novel therapeutic concept to limit breast cancer-induced osteolytic disease.

Elevated miR-218-5p expression has previously been associated with estrogen receptor- positive breast tumors indicating a possible role in certain breast cancer phenotypes [31]. Furthermore, elevated miR-218-5p expression is a promising diagnostic and prognostic biomarker of human chondrosarcoma [32]. In contrast, during early osteosarcoma miR-218-5p is down-regulated [33] and in other cancers miR-218-5p was reported to function as a tumor suppressor through regulation of the Slit-Robo pathway [34]. While Slit2 is expressed in metastatic breast cancer cells, it is associated with metastasis to brain but not to bone [35] whereas both estrogen and Wnt signaling are linked to bone metastasis [36]. In particular, Wnt signaling influences bone metastasis through autocrine mechanisms affecting processes that include survival and proliferation, and paracrine mechanisms by modulating the bone microenvironment for efficient colonization and outgrowth of tumor cells [25, 37]. It is appreciated that the power of miRNAs in regulating biological processes in a temporal-spatial manner is influenced by the cellular environment and the activity of signaling pathways. For example, miRNAs can act as tumor suppressors or oncogenes depending on the cellular context and cell specific signaling pathways and targets [9, 12]. This functional ambivalence is understandable in view of the roles of miRNAs in feed-back and feed-forward mechanisms that control normal developmental signaling pathways. Our findings suggest that regulation of Wnt signaling, which is an important pathway in bone metastasis, is a prominent mechanism for miR-218-5p in breast cancer metastasis.

Although other miRNAs have been identified in regulating Wnt signaling and miR-218 has been associated with other cancers, our study defines a unique functional activity of miR-218-5p in promoting metastatic tumor growth of triple negative breast cancer cells in bone and further exacerbating the osteolytic bone disease. miR-218-5p directly downregulates two Wnt inhibitors accompanied by increased Wnt activity and markedly elevated expression of Wnt target genes CyclinD1, LEF1 and TCF4. These novel findings are consistent with clinical observations. For example, high expression of CyclinD1 predicts for poor prognosis in breast cancer patients [38]. Functionally, CyclinD1 enhances cell proliferation [39]. Thus, the cell-autonomous impairment of proliferation by antimiR-218-5p may be due to downregulation of CyclinD1. Importantly, more prominent differences were observed in tumor growth and cancer cell proliferation *in vivo*, suggesting additional mechanisms at play. Indeed, several metastasis-related genes were down-regulated upon inhibition of miR-218-5p in breast cancer cells, including CXCR4, a crucial cytokine supporting breast cancer homing to and growth in bone supporting the importance of antimiR-218-5p in impairing breast cancer growth in the bone environment [40]. For example, antimiR-218-5p impaired cancer-induced bone destruction thereby reducing the release of several growth factors from the bone environment upon cancer-induced osteolysis which consequently supports the vicious cycle of tumor growth [3, 4]. Thus both the cell-autonomous and bone environment-mediated mechanisms contribute to reduced tumor growth by antimiR-218.

Breast cancer cells exhibit properties of osteoblast, reflected by expression of bone matrix proteins, a process referred as osteomimicry. BSP and OPN are prominent, mineral-associated proteins in the extracellular matrix of bone that have been implicated in the metastatic activity of cancer cells and that may have a role in targeting metastatic cells to bone [41]. Interestingly, antimiR-218-5p decreased the expression of both BSP/IBSP and OPN/SPP1. Thus, the miR-218-5p/Wnt axis may support osteomimicry of bone metastatic breast cancer cells by promoting expression of osteoblast-related ECM proteins and paracrine factors under normal physiological conditions in osteoblasts (i.e., during skeletal development and early bone formation) and under pathological conditions in metastatic breast cancer cells (i.e., in adult patients which homeostatically regulate bone mass). Beyond its function in osteoblasts, miR-218-5p is required for normal development of several additional cell types that include motor neurons [42, 43]. Although we did not observe obvious side-effects of miR-218-5p *in vivo*, it cannot be excluded that systemic inhibition of miR-218-5p can cause adverse effects in other tissues. Therefore, it will be important in the future to investigate novel tools to target delivery of small RNA specifically to tumor cells.

Progression of breast cancer to the stage of bone metastasis has a devastating clinical outcome. PTHrP is a key cytokine secreted from tumor cells that promotes metastatic bone destruction by activating osteoclasts. Recently, activation of Wnt signaling in metastatic breast cancer cells was shown to induce expression of PTHrP through the Gli1 transcription factor [25]. Our findings link miR-218-5p with the Wnt-PTHrP cascade and demonstrate the functional relevance of the pathway in osteoclast differentiation. Of translational relevance, antagonizing miR-218-5p in cancer cells strongly reduced Rankl expression in osteoblasts through PTHrP and perhaps other secreted factors, leading to inhibition of osteoclast differentiation *in vitro* and *in vivo*. Although approaches to replace or inhibit miRNAs have been investigated in the context of other tumors, only a limited number of miRNAs have been associated with breast cancer-induced osteolytic disease. Promising results have been obtained by targeting osteoclasts with miR-141 and miR-219 [44] or breast cancer cells with miR-135 and miR-203 [16]. Both approaches cause a reduction of the osteolytic disease. While these results provide encouraging support for the development of miRNA-based therapeutic interventions, both studies [16, 44] used miRNA replacement therapy. Repressing endogenous miRNA function using synthetic, sequence-specific antisense miRNA-inhibitors (anti-miR) is a complementary and equally effective option for reducing tumor properties [45]. Our study establishes a novel anti-miR-based approach to limit the osteomimetic properties of disseminated tumor cells and osteolytic disease, which can open new dimensions for the development of translational strategies for miRNA-mediated treatment of metastatic bone disease.

## MATERIALS AND METHODS

### Tissue samples

Tissue biopsies derived from primary breast cancer were purchased from Amsbio. Tissue biopsies from bone metastases of breast cancer patients and from healthy age-matched women were obtained from the archives of the University Medical Center Hamburg-Eppendorf, Germany, following institutional guidelines. Tissue samples were evaluated independently by two expert pathologists. All studies using human samples were carried out in accordance with the declaration of Helsinki.

### Cell culture

The human mammary epithelial cell line (ER-; MCF-10A) and the breast cancer cell lines MCF-7 (ER+) and MDA-MB-231-a (ER-; hereafter MDA-MB-231) were purchased from ATCC. The MDA-MB-231-b subclone was kindly provided by Dr. Theresa Guise. Both cell lines are highly metastatic triple negative (ER-, PR-, HER-) breast cancer cells. Generation and characterization of the ER- MCF-10A-derived pre-malignant MCF-10AT1 and malignant MCF10CA1a cell lines has been previously described [21, 22].

MCF-10A cells were cultured in MEGM medium (Lonza) supplemented with 100 ng/ml cholera toxin. MCF-7 cells were maintained in D-MEM (high Glucose) (Lonza) supplemented with 10% Fetal Bovine Serum (FBS, Atlanta) and 1% Penicillin/Streptomycin (Gibco). MDA-MB-231 cells were cultured in alpha-MEM (Lonza), 10% FBS and 1% Penicillin/Streptomycin. All cell lines were validated at the Vermont Cancer Center DNA Analysis Facility by STR DNA fingerprinting using the Promega GenePrint® 10 System according to manufacturer's instructions (Promega #B9510). The STR profiles were compared to known ATCC fingerprints (ATCC.org), and to the Cell Line Integrated Molecular Authentication database (CLIMA) version 0.1.200808 (http://bioinformatics.istge.it/clima) [23]. The STR profiles of all cell lines matched (>85%) known DNA fingerprints. To inhibit Wnt signaling MDA-MB-231 cells were treated with 30 μM small molecule inhibitor CCT036477 (Enzo Life Sciences) [24]. To activate Wnt signaling, cells were incubated with lithium chloride solution (Sigma-Aldrich) at a final concentration of 40 mM [25]. To collect conditioned medium (CM), MDA-MB-231 cells were seeded at 80% confluence in complete medium. Cells were serum starved for 24 h in 2% FBS prior collection of the CM. CM was centrifuged to remove cell debris and used immediately or stored at -80°C.

Long bone osteoblasts were isolated from the femora and tibiae of 8-10-week old wild type C57/Bl6 mice. After removing the muscles in sterile PBS, bone marrow was flushed out and bones were cut into small pieces. Bone pieces were digested with 0.1% collagenase for 2 hours at 37°C and plated in α-MEM containing 10% FBS and 1% P/S. Outgrowing osteoblasts were trypsinized after one week and cultured until confluence (2-3 weeks). Bone marrow macrophages (BMMs) were isolated from the bone marrow of 8-week old wild type mice. Non-adherent cells were collected after 3 hours of incubation on plastic and cultured with Macrophage colony-stimulating factor (MCS-F; 100 ng/ml, Peprotech). For osteoblast-osteoclast co-cultures, long bone osteoblasts were plated on 96-well plates and stimulated with Vitamin D_3_ and prostaglandin E. One day later, BMMs were plated over osteoblasts. Cultures were terminated after 5 days, fixed and stained with Tartrate-resistant acid phosphatase (TRAP) for 10 min at 37°C. All reagents for TRAP solution (Naphthol-ASMX-Phosphate, Fast Red Violet LB-Salt and N, N-Dimethylformamid) were purchased from Sigma. TRAP-positive cells with a minimum of three nuclei were considered as osteoclasts and counted using the Osteomeasure system (Osteometrics).

### Immunohistochemistry

Human tissue biopsies and mouse bones and lungs were fixed in 4% Formalin/PBS. Bones were decalcified in 4% Na-EDTA solution at pH 7.4 for 2 weeks. Tissues were dehydrated, embedded in paraffin and cut. Consecutive 4 μm thick sections were analyzed by immunohistochemistry using antibodies against β-Catenin (Cell Signaling), Ki-67 (Dako), PTHrP (Cell Signaling) and HLA class 1 ABC (Abcam) with positive and negative controls following established protocols [26]. Antigen retrieval was performed using citrate buffer at pH 6.0. Vectastain (Vector Laboratories) and DAB+ (Dako) systems were used for detection. TRAP solution was prepared as described and sections were stained for 10 min at 37°C. Ki-67-positive cells in bones and HLA-positive metastatic nodules in mouse lungs were quantified using the Osteomeasure system.

### Transfections

MDA-MB-231 cells were plated in 6-well plates and transfected at 70-80% confluence with *mir*Vana™ miRNA mimics and inhibitors (Ambion) at a final concentration of 50 nM in OPTI-MEM using Oligofectamine Reagent (Invitrogen) according to manufacturer's instructions. Fresh culture medium containing 20% FBS was added after 4 h. Cells were used for functional studies or harvested for RNA and protein analyses as described.

For the 3′ UTR assays, the 3′UTRs of *SOST* and *SFRP2* were cloned into a pMIR-Reporter Luciferase Plasmid (Applied Biosystems) to obtain Luc-*SOST*-3′UTR and Luc-*SFRP2*-3′UTR reporter plasmids [20]. Cells were transfected with miRNA mimic and inhibitor oligonucleotides using oligofectamine as described above. On the following day, cells were transfected with 500 ng of 3′UTR plasmids each along with 50 ng Renilla luciferase plasmid (Promega) using Xtremegene9 (Roche) following manufacturer's instructions. Luciferase assays were performed using the Dual Luciferase Reporter Gene Assay System (Promega) according to instructions provided by the manufacturer. Lentivirus clones control (PMIRH000PA-1), miRNA-218 (PMIRH218-2PA-1) and anti-miR Lentivirus (miRZip-218 anti-miR, MZIP218-PA-1) were purchased from System Biosciences and infected as described previously [27].

### Total RNA extraction and gene expression analysis

Total RNA was isolated using Trizol reagent (Invitrogen) and purified with Zymo RNA purification kit (Zymo) according to manufacturer's instructions. cDNA was synthesized from 1 μg of total RNA using Supercript RT kit (Invitrogen) following the protocol provided by the manufacturer. Quantitative real-time PCR (qRT-PCR) was performed with the 7300 sequence detection system (Applied Biosystems/Roche) using SYBR^®^ Green Master Mix (Applied Biosystems). After normalization to Glyceraldehyde 3-phosphate dehydrogenase *(GAPDH)* mRNA, relative expression levels and fold induction of each target gene were calculated using the comparative C_T_ (ΔΔCT) method.

### MiRNA extraction and expression analysis

Total RNA was isolated from cells and purified as described above. RNA extraction from paraffin embedded tissue samples was performed using the High Pure FFPE RNA Micro-kit (Roche) according to manufacturer's instructions [26]. The QuantimiR-kit (SBI System Biosciences) was used to add a polyA tail to the small RNAs for cDNA synthesis (SBI System Biosciences) according to manufacturer's guidelines. Relative miRNA expression was determined by SYBR Green detection (Applied Biosystems) using a universal reverse primer and a specific forward primer designed for miR-218-5p [20]. U6 expression was used as internal control. The relative miRNA expression was calculated using the ΔΔCT method.

### ELISA

Conditioned medium was collected from MDA-MB-231 cells transfected with miR-218-5p mimics and inhibitors as described above. PTHrP was quantified in the conditioned medium using ELISA (Lifespan Biosciences).

### Proliferation assay

MDA-MB-231 cell proliferation was determined by the CellTiter MTS assay (Promega). Cells were seeded in 96-well plates at a density of 3000 cells/well before performing the MTS assay according to manufacturer's instructions.

### Migration assays

Cell migration was determined using a wound healing assay. For this purpose, MDA-MB-231 cells were plated in 6-well plates, transfected with miRNA mimics or inhibitors as described and cultured to 90-100% confluence. A wound was then applied with a pipette tip and closure of the wound was monitored by microscopy. The width of the wound was quantified using the ImageJ program.

### Intratibial injection of miRNA-transfected breast cancer cells

All animal studies were conducted in accordance with approved Institutional Animal Care and Use Committee protocols following the ethical guidelines of the NIH Guide for the Care and Use of Laboratory Animals. Severe combined immunodeficient (SCID) mice were obtained from the Jackson Laboratory. MDA-MB-231 cells stably expressing luciferase (MDA-MB-231-*luc*) were transfected with miR-218-5p or antimiR-218-5p and the control oligonucleotides as described. After 24 h, cells were counted and suspended in sterile PBS (100,000 cells/100 μl). Female SCID mice (6-week old) were anesthetized by intraperitoneal (i.p.) injection of Ketamine/Xylazine (Ketamine: 100 mg/kg body weight; Xylazine: 10mg/kg body weight). The knee of the right hind limb was flexed and a short incision was made over the knee. A 30-gauge needle on a tuberculin syringe was then inserted into the intramedullary space of the tibia, followed by the careful injection of 100 μl of MDA-MB-231-*luc* cell suspension. A minimum of 6 mice were used per group.

### Monitoring tumor growth and osteolysis

Bioluminescence imaging (Xenogen) was used to follow the tumor growth in the bone. For this purpose mice were anesthetized using isoflurane followed by the injection of D-luciferin (i.p.) at a dose of 150 mg/kg per body weight. The animals were placed on the warm (37°C) IVIS imaging stage and bioluminescent images were acquired for each mouse using the IVIS Imaging System (Perkin Elmer). Analyses were performed using the LivingImage software (Xenogen) by measuring the photon flux within a region of interest drawn around the bioluminescence signals. Blank regions of interest were also measured for each scan and subtracted from each tumor photon flux for normalization.

Tibiae were scanned in air aligned axially using a micro CT scanner (Scanco Medical μCT 40) at 70kV_p_, 114μA and a resolution of 10μm.

### miRNA target prediction and pathway analysis

Putative targets of miR-218-5p were identified in IPA (www.ingenuity.com, QIAGEN, Germantown, MD) using default parameters and restricted to downstream molecules within the miR-218-5p interaction network. Ingenuity canonical pathways related to osteogenesis were identified based on significant enrichment. miR-218-5p targeted genes were mapped onto these pathways.

### Statistical analysis

Normal distribution of the data was tested using the D'Agostino-Pearson omnibus test. Comparisons between groups were analyzed using one-way ANOVA with Tukey's post-hoc tests.

## SUPPLEMENTARY FIGURES AND TABLES


