# Supplementary material for: Antagonizing miR-218-5p attenuates Wnt signaling and reduces metastatic bone disease of triple negative breast cancer cells
Source: Oncotarget. 2016 Oct 12;7(48):79032–46. doi: 10.18632/oncotarget.12593 (PMC5346696; doi:10.18632/oncotarget.12593)
Supplement: Supplementary file 1 [file oncotarget-07-79032-s001.pdf]

Antagonizing miR-218-5p attenuates Wnt signaling and reduces metastatic bone disease of triple negative breast cancer cells

SUPPLEMENTARY FIGURES

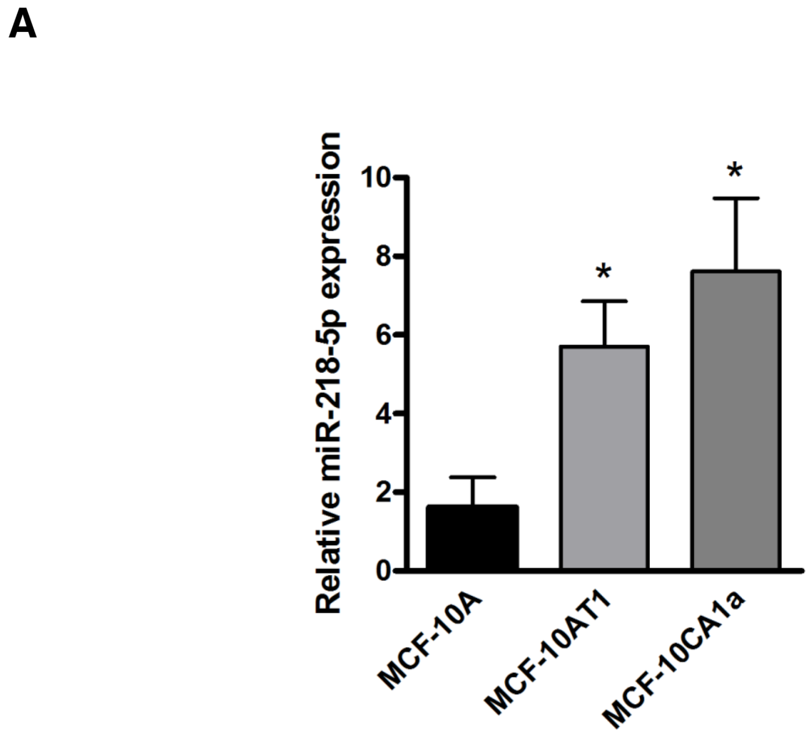

Supplementary Figure S1: miR-218-5p expression is increased in malignant epithelial cells. Expression of miR-218-5p was determined in non-malignant epithelial MCF-10A cells, premalignant MCF-10AT1 cells and malignant MCF-10CA1 cells by qRT-PCR. N = 3 independent experiments. Three biological replicates from different passages were used for each cell line. Mean values ± SEM, *$p<0.05$.

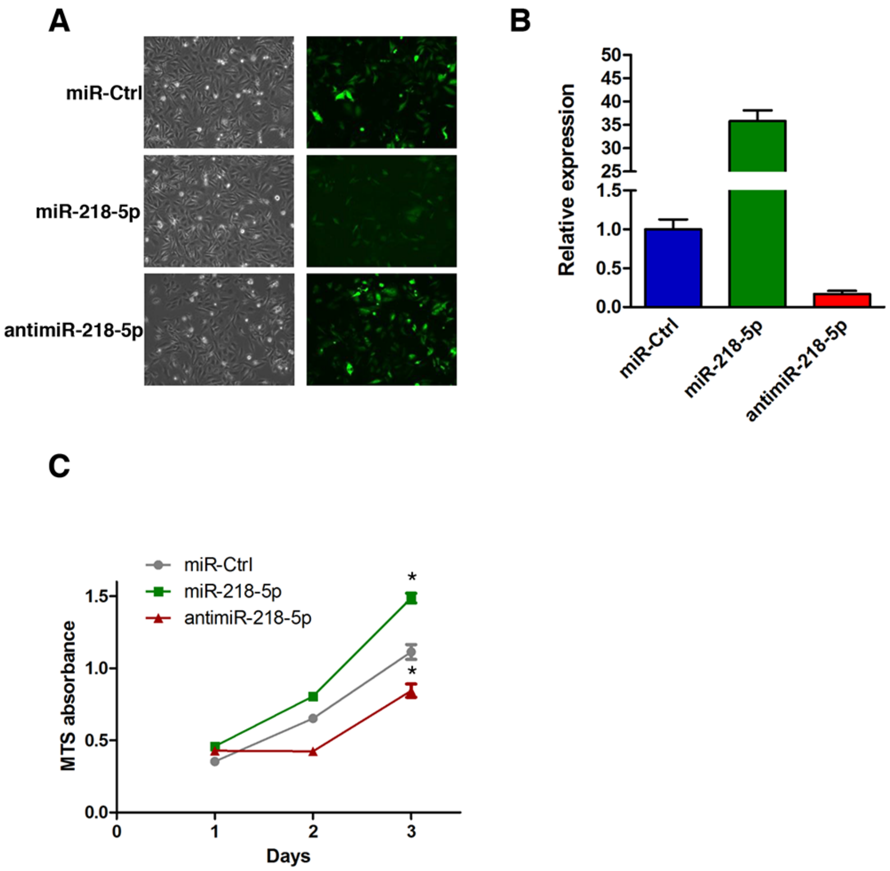

Supplementary Figure S2: Stable expression of miR-218-5p or antimiR-218-5p alters breast cancer cell proliferation. **A.** MDA-MB-231 cells were infected with lentiviral vectors containing GFP and miR-218-5p or antimiR-218-5p. Cell morphology was determined by phase contrast microscopy. GFP signal was observed with epifluorescence microscopy. **B.** MDA-MB-231 cells were transfected with control, miR-218-5p or antimiR-218-5p oligonucleotides. miR-218-5p expression was determined by qRT-PCR. **C.** Cell proliferation was analyzed in lentivirus infected cells with an MTS Assay. N= 3 independent experiments. Mean values ± SEM, *$p<0.05$.

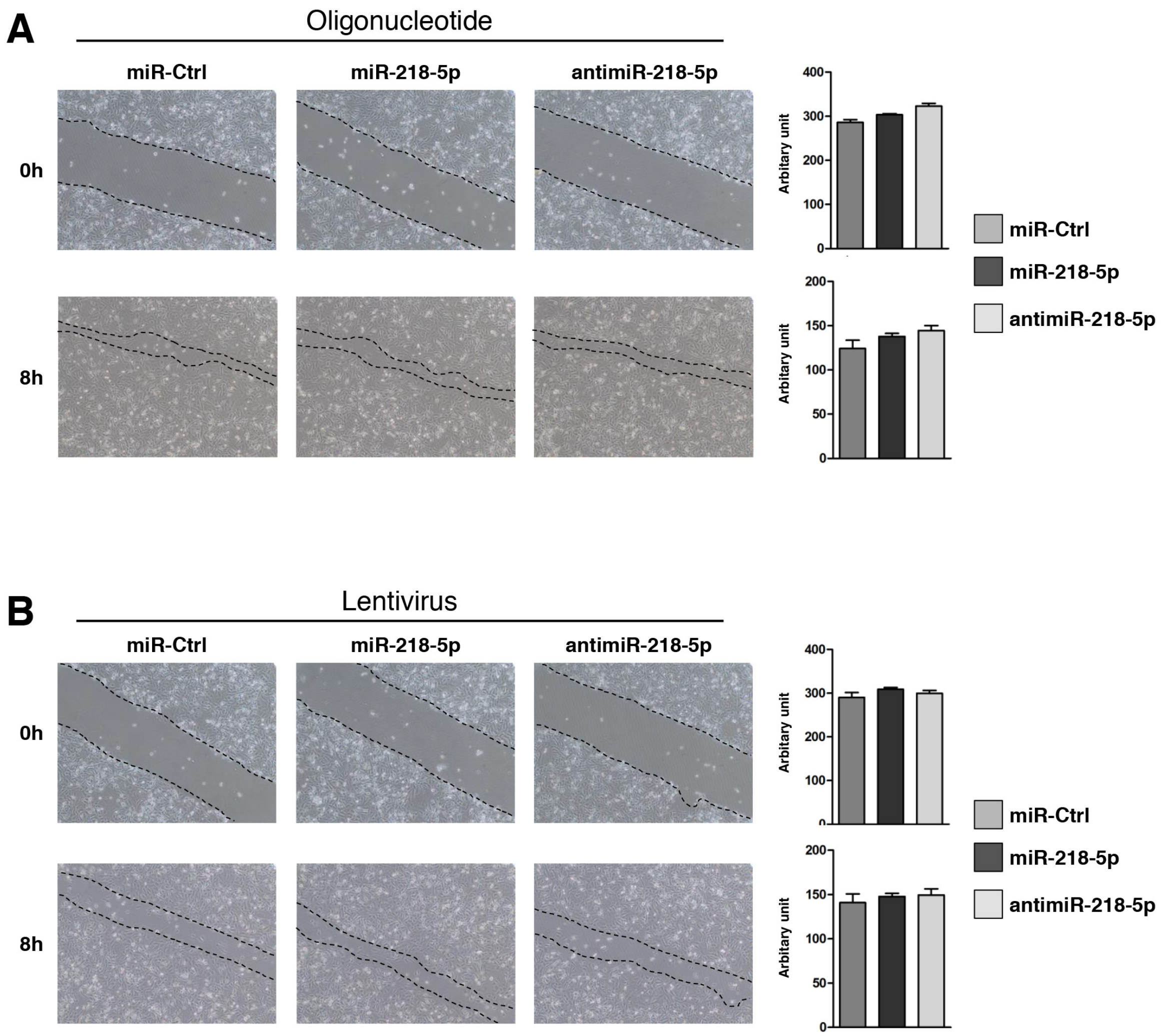

Supplementary Figure S3: miR-218-5p does not affect breast cancer cell migration. A. Wound healing assay using MDA-MB-231 cells transfected with miR-218-5p or antimiR-218-5p oligos. Pictures were captured immediately and 8 hours after the would was applied and wound width was quantified using ImageJ. **B.** Migration of MDA-MB-231 cells stably expressing miR-218-5p or antimiR-218-5p. N= 3 independent experiments. Mean values ± SEM.

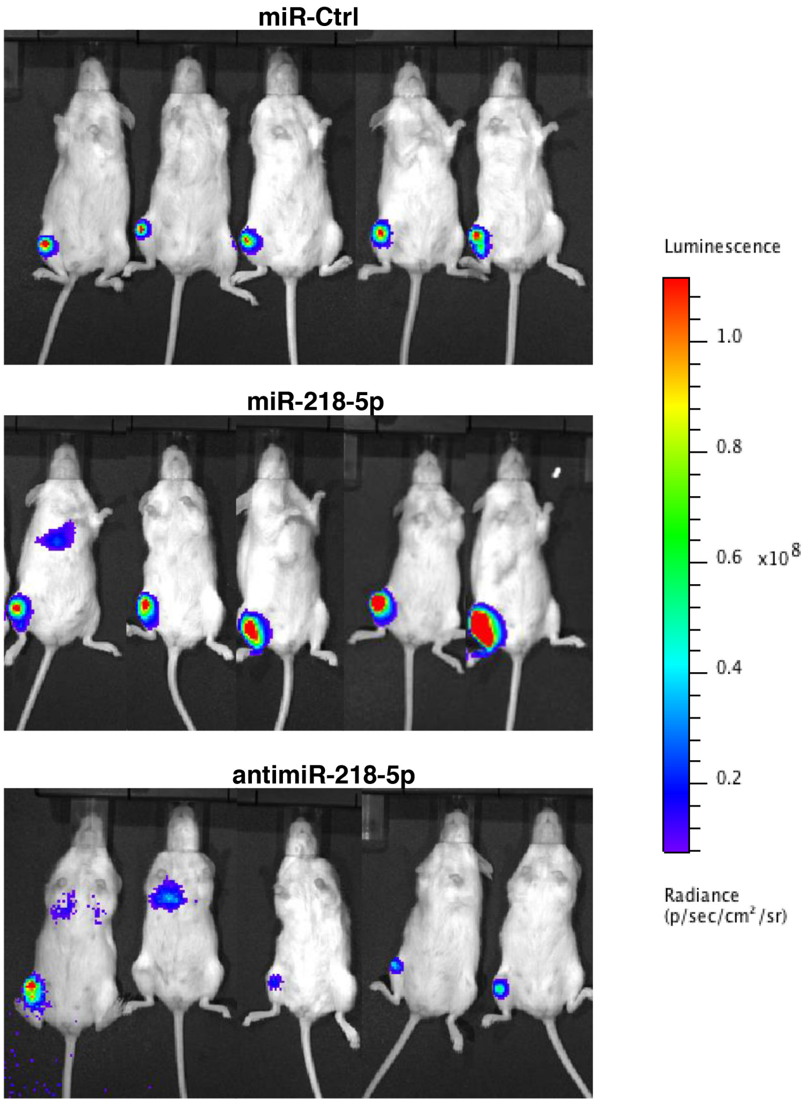

Supplementary Figure S4: AntimiR-218-5p treatment reduces tumor growth in bone. MDA-MB-231 cells stably expressing luciferase were transfected with miR-218-5p, antimiR-218-5p or control miRNA (miR-Ctrl) oligonucleotides and transplanted into the tibiae of immunocompromised mice. Tumor growth was visualized after four weeks by bioluminescence imaging.

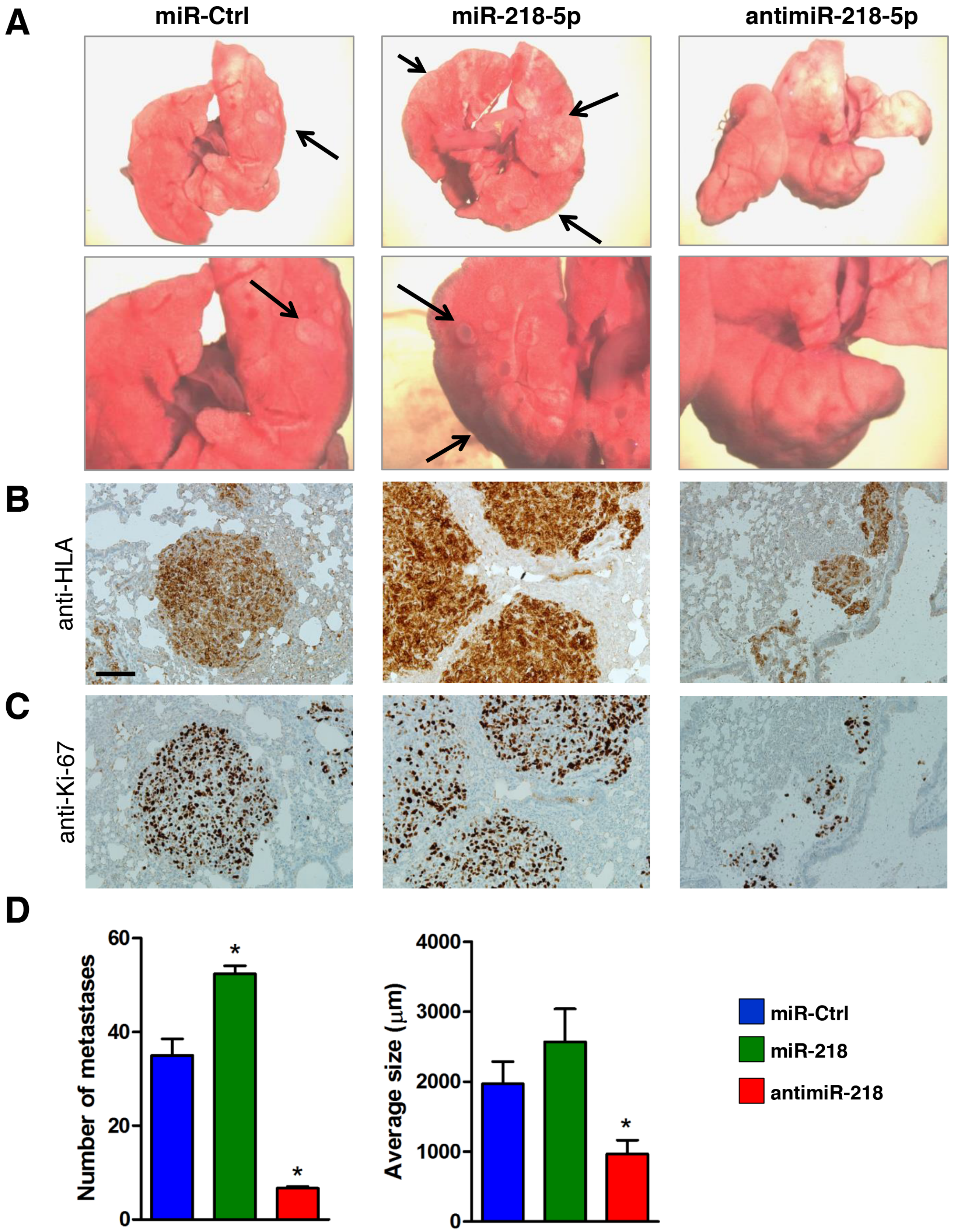

Supplementary Figure S5: AntimiR-218-5p reduces lung metastasis. A. Images were taken from mouse lungs using a stereomicroscope (top panels). Detection of metastatic nodules indicated by arrows in (A), originating from implanted human breast cancer cells using a immunohistochemical staining against human specific anti-HLA **B.** and Ki-67 **C.** Scale bar indicates 200 μm. **D.** The number and average size of metastatic nodules was quantified with Osteomeasure. N=6 mice/group. Mean values ± SEM, *p<0.05.

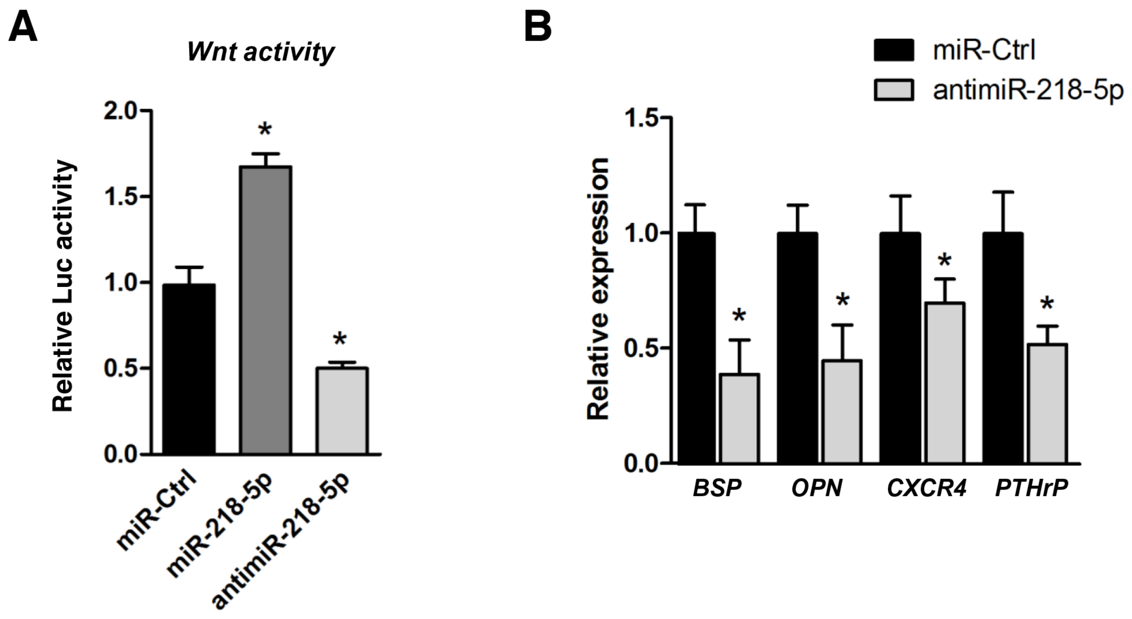

Supplementary Figure S6: AntimiR-218-5p reduces Wnt activity and the expression of metastasis-related Wnt target genes. A. Wnt signaling was determined in MDA-MB-231 cells stably expressing miR-218-5p or antimiR-218-5p by TopFlash Assay. **B.** Expression of Wnt target genes was analyzed by qRT-PCR in MDA-MB-231 cells transfected with antimiR-218-5p. N= 3-4 independent experiments. Mean values ± SEM, *p<0.05.
